# A novel strategy to attenuate porcine reproductive and respiratory syndrome virus by inhibiting viral replication in the target pulmonary alveolar macrophages via hematopoietic-specific miR-142

**DOI:** 10.1186/s44280-023-00002-2

**Published:** 2023-03-30

**Authors:** Shaoyuan Tan, Weixin Wu, Xinna Ge, Yongning Zhang, Jun Han, Xin Guo, Lei Zhou, Hanchun Yang

**Affiliations:** 1 Department of Infectious Diseases, St. Jude Children’s Research Hospital, Memphis, TN 38105 USA; 2 National Key Laboratory of Veterinary Public Health Security, College of Veterinary Medicine, China Agricultural University, Beijing, China; 3 Key Laboratory of Animal Epidemiology of Ministry of Agriculture and Rural Affairs, College of Veterinary Medicine, China Agricultural University, Beijing, China

**Keywords:** PRRSV, Virulence attenuation, miR-142

## Abstract

Porcine reproductive and respiratory syndrome virus (PRRSV) is an economically important pathogen for the global pork industry. Although modified live virus (MLV) vaccines are commonly used for PRRSV prevention and control,  they still carry a risk of infecting the host and replicating in target cells, thereby increasing the likehood of virus recombination and reversion to virulence. In this study, we inserted the target sequence of miR-142 into the nsp2 hypervariable region of PRRSV to inhibit viral replication in its host cells of pigs, with the aim of achieving virus attenuation. The chimeric virus RvJX-miR-142t was successfully rescued and retained its growth characteristics in MARC-145 cells. Furthermore, it did not replicate in MARC-145 cells transfected with miRNA-142 mimic. We also observed limited replication ability of RvJX-miR-142t in pulmonary alveolar macrophages, which are the main cell types that PRRSV infects. Our animal inoculation study showed that pigs infected with RvJX-miR-142t displayed less severe clinical symptoms, lower viremia titers, lighter lung lesions, and significantly lower mortality rates during the first 7 days post-inoculation, in comparison to pigs infected with the backbone virus RvJXwn. We detected a partially deletion of the miR-142 target sequence in the RvJX-miR-142t genome at 14 dpi. It is highly possible that the reversion of viral virulence observed in the later timepoints of our animal experiment was caused by that. Our study provided a new strategy for attenuating PRRSV and confirmed its effectiveness. However, further studies are necessary to increase the stability of this virus under host selection pressure.

## Introduction

Porcine reproductive and respiratory syndrome (PRRS) is clinically characterized as reproductive failure in breeding pigs and respiratory distress in pigs of all ages, leading to elevated mortality and poor growth performance, particularly in weaning and nursery herds [1]. PRRS is a signification viral disease for the global pig industry, with important economic implications. A recent study on the economic impacts of PRRS in China revealed that direct economic losses in a PRRS outbreak farm could reach 1424.37 RMB per sow and could be further exacerbated by an increased feed price [2]. In 2006, an epidemic of atypical PRRS caused by the emerging highly pathogenic PRRS virus (HP-PRRSV) represented by JXA1 or JXwn06 with a unique 30-amino-acid deletion in its nsp2-coding region, result in significant economic losses to the pork industry was pandemic in China [3, 4]. HP-PRRSV became the predominant strain in China less than a year after its initial outbreak and has since been widely circulated for more than eight years [5, 6]. Initially, a killed vaccine based on the JXA1 strain was launched to prevent and control HP-PRRSV outbreak; however, its effectivenness was unsatisfactory. Consequently, a series of HP-PRRSV-derived modified live virus (MLV) vaccines were developed and approved for use in the field. Although the usage of MLV can provide significant homologous protection against the HP-PRRSV and reduce the mortality in outbreak herds, there are concerns about its safety, as many field isolates with identical nucleotide sequences to the vaccine viruses have been frequently discovered [5]. The current attenuation process of PRRS MLV can be achieved in several ways, including continuous passaging in nontarget cells, DNA shuffling, construction of chimeric virus, and codon pair deoptimization. These approaches mainly reduce the replication capability of the virus in pulmonary alveolar macrophages (PAMs), which are the primary target cells of PRRSV [7]. However, the MLV strains can still infect the host and replicate in the target cells, which increases the risk of recombination with field strains and reversion to virulence. Our recent studies have confirmed that the HP-PRRSV strain JXwn06-P80 attenuated via passage in MARC-145 cells could regain its fatal virulence after increasing its adaptability in PAMs [8]. Additionally, MLV infection in PAM can also cause varying levels of immunosuppression, which is another concern regarding its safety. Therefore, eliminating the replication capability of the virus in PAMs but remaining its immunogenicity would be a good strategy in designing novel PRRSV vaccines.

PRRS virus (PRRSV), the causative agent of PRRS, is an enveloped, single-stranded positive-sense RNA (+ ssRNA) virus. It is divided into two species within the *Arteriviridae* family, *Betaarterivirus suid* 1 (PRRSV 1, typically called European type) and *Betaarterivirus suid* 2 (PRRSV 2, typically called North American type) [9]. The PRRSV genome is approximately 15 kb that has at least 12 open reading frames (ORFs). ORF1a and ORF1b encode the polyproteins pp1a and pp1ab, which are further processed into 16 nonstructural proteins (nsps) responsible for viral transcription and replication. The remaining genome at the 3’ termini encodes the viral structural proteins [10]. The nonstructural protein 2 (nsp2) coding region is one of the most variated regions with mutations, deletions, and insertions that occur naturally, suggesting that the nsp2 region of PRRSV can tolerate the insertion of foreign genes. The genetic flexibility of nsp2 has been used to construct a series of recombinant viruses with foreign tags such as green fluorescent protein (GFP) [11], HiBiT subunit of neo luciferase [12], Myc [13], and immunodominant B-cell epitope of Newcastle disease virus (NDV) [14].

MicroRNA (miRNA) is a type of non-coding RNA (ncRNA), which forms an extensive class of ncRNAs that regulate the expression of genes at the post-transcriptional level [15]. In the cytoplasm, pre-miRNAs are processed by Dicer, an RNAse III enzyme, into imperfect dsRNA duplexes that contain both mature and complementary miRNA strands [16]. The strand is loaded into an RNA-induced silencing complex (RISC), thus becoming a mature miRNA, and RISC can impose a translational block of the target mRNA [17]. Previous reports have found many miRNAs to inhibit virus replication by regulating the expression of the viral receptor, antivirus factors, or even by directly binding to the viral genome [18], such as miR-181 mimics, which can specifically bind to ORF4 to inhibit PRRSV replication [19]. As miRNAs emerge as important regulators of protein expression during tissue development and homeostasis, some miRNAs only express in specific tissues or cell types [20, 21]. For example, the miRNA-122 (miR-122) was one of the first examples of a tissue-specific miRNA highly expressed in the liver [22]. Additionally, five miRNAs, including miR-142, miR-144, miR-150, miR-155, and miR-223, are highly specific for hematopoietic cells [21]. Thus, the tissue-specific miRNAs could be used to control viral tropism by inserting their target sequences into the virus genome to restrict replication exclusively in this cell population [23]. This strategy has been applied to poliovirus [24] and influenza A virus [25]. Meanwhile, our early transcriptome data of PAMs infected with PRRSV indicated that the expression level of miR-142 in PAMs was stable and wasn’t regulated by PRRSV infection [26]. In addition, there is no miR-142 expression in MARC-145 cells. These studies inspire us to use hematopoietic-cell-specific miRNAs to inhibit PRRSV replication in its target cells to achieve virus attenuation.

The objective of this study is to generate a vaccine candidate that can grow rapidly in MARC-145 cells for vaccine preparation but has limited replication capability in PAMs for reduced pathogenicity. We incorporated the miR-142 targeted sequence into the PRRSV nsp2 coding region and rescued the chimeric virus by using reverse genetic operation. Then replication ability and pathogenicity of the rescued chimeric virus were systematically assessed both in vitro and in vivo. The results indicated that the chimeric virus RvJX-miR-142t had limited replication ability in PAMs and lower pathogenicity in pigs compared with its parental backbone virus RvJXwn. However, under selective pressure from the hosts, the miR-142 target sequence was partially deleted in virus genomes extracted from clinical samples of 14 days post-inoculation, resulting in the reversion of virulence observed clinically.

## Results

### The viral proliferation ability of RvJX-miR-142t was obtained in MARC-145 cells, but reduced in PAMs

**Fig. 1:**
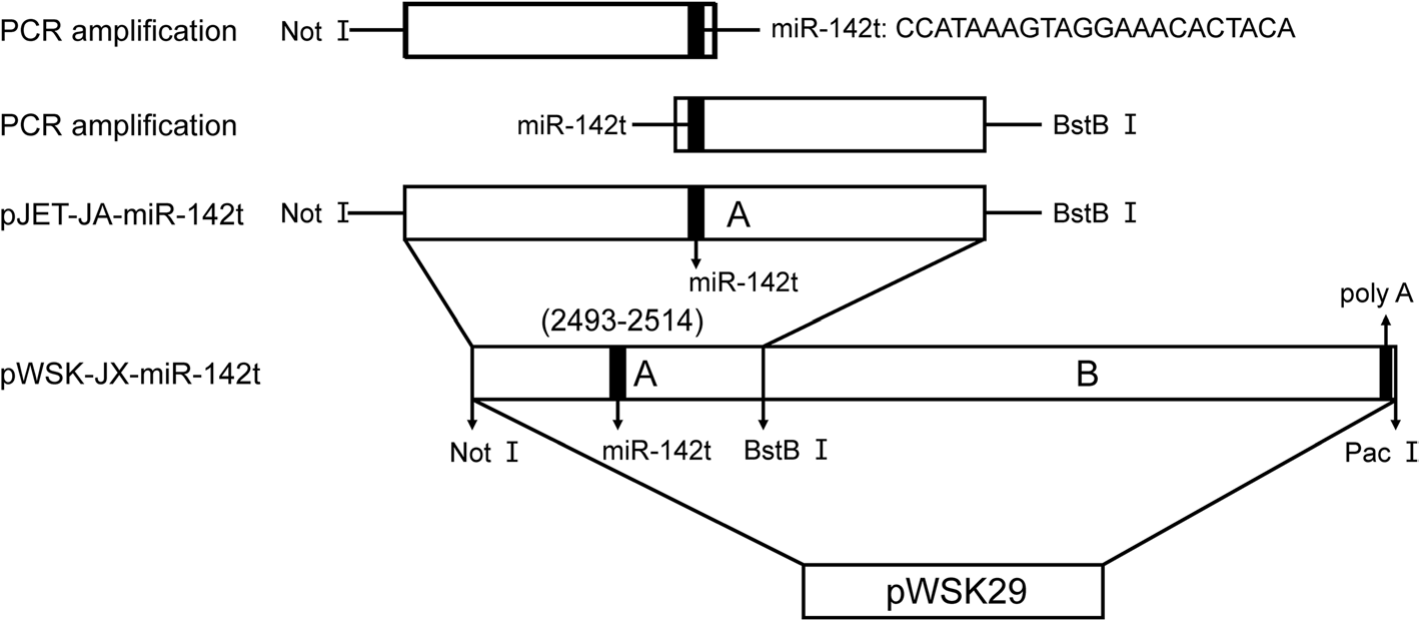
The strategy for the construction of full-length cDNA clones. The numbers refer to nucleotide positions within the genome of JXwn06. Capital letters (**A**, **B**) represent two fragments of the JXwn06 genome according to the unique restriction enzyme cleavage sites in viral cDNA

**Fig. 2:**
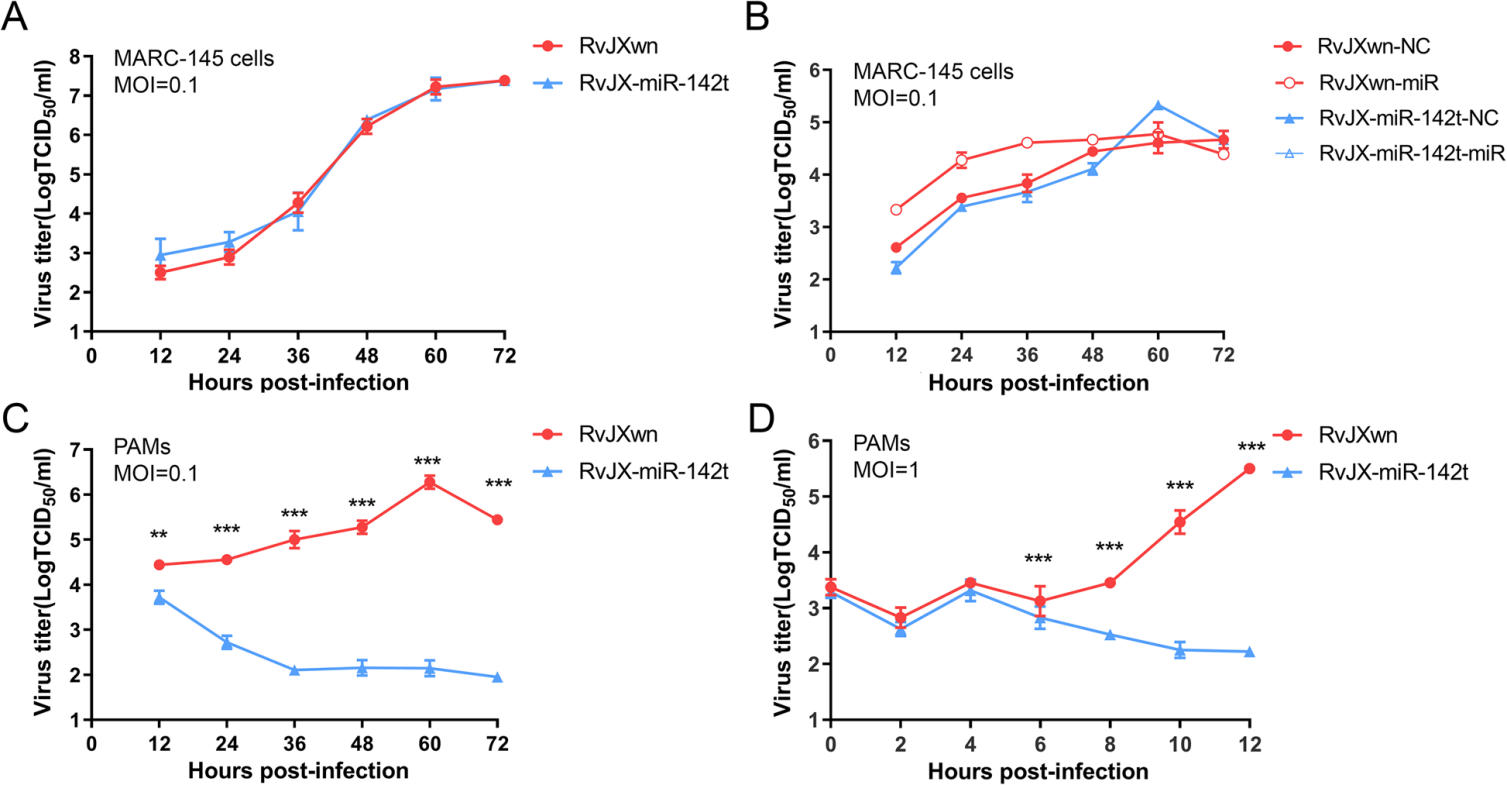
The growth kinetics of rescued PRRSV in MARC-145 cells (**A**), MARC-145 cells transfected with miR142 mimic (**B**) and PAMs (**C** and **D**). The data are shown as means ± SD (error bars). Asterisks indicate a significant difference in the virus titers between RvJXwn and RvJX-miR-142t. (**, *p* < 0.01; ***, *p* < 0.001)

The recombinant virus with miR-142 target sequence was successfully rescued and was named RvJX-miR-142t (Fig. 1). To test its stability, RvJX-miR-142t was serially passaged in MARC-145 cells for 10 rounds, and its genome was sequenced at P5 and P10. The results showed there was no mutation, insertion, or deletion in the region inserted with miR-142t. The growth kinetics of RvJX-miR-142t and RvJXwn were evaluated by infecting MARC-145 cells, MARC-145 cells transfected with miR-142 mimic, and primary PAMs. The results showed that RvJX-miR-142t had similar growth kinetics to RvJXwn in MARC-145 cells (Fig. 2A), indicating that the insertion of miR-142t did not affect its replication ability in MARC-145 cells. However, in MARC-145 cells transfected with miR-142 mimic, the titers of the recombinant virus RvJX-miR-142t couldn’t be detected at all time points, indicating its proliferation ability was severely inhibited (Fig. 2B). For the multi-step growth curve on PAMs (MOI = 0.1), the parental virus RvJXwn showed a normal trend of rising and reached its peak titer of 10^6 TCID_50_/mL at 60 h post-inoculation (hpi). Meanwhile, virus titer of the RvJX-miR-142t group continuously declined from 12 to 72 hpi (Fig. 2C). For the single-step growth curve on PAMs (MOI = 1), the virus titer of RvJXwn kept rising after 6 hpi, but the virus titer of RvJX-miR-142t continued to decline after 4 hpi (Fig. 2D). These results above indicated that the miR-142t sequence inserted into the PRRSV genome could be targeted by miR-142, causing the inhibited replication of RvJX-miR-142t in cells expressing miR-142, such as PAMs.

### The pathogenicity of RvJX-miR-142t decreased significantly *in vivo* before 15 dpi

**Fig. 3:**
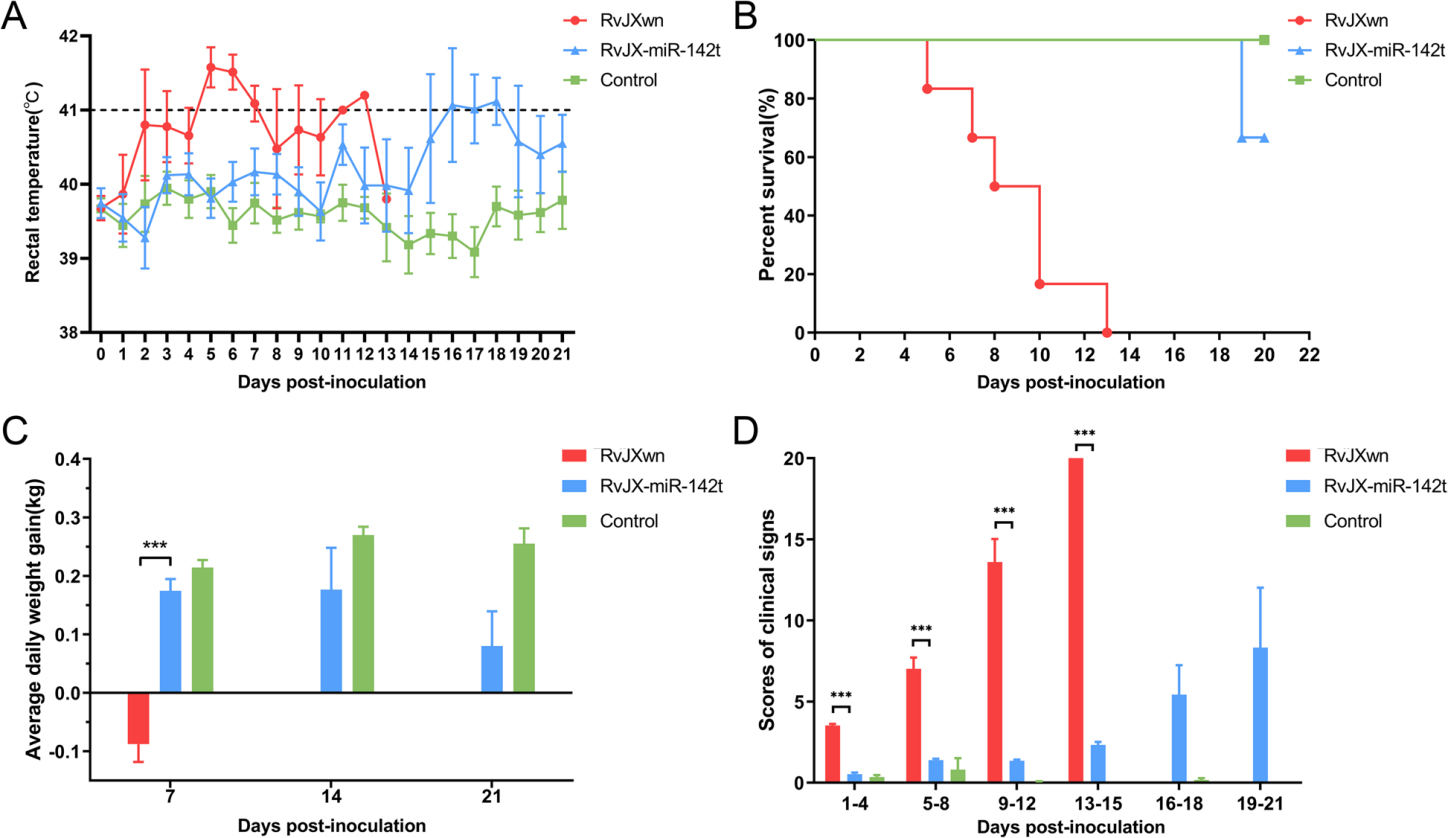
The clinical symptoms and mortality of inoculated pigs. The rectal temperatures (**A**), survival rate (**B**), average daily weight gain (ADG) (**C**), and clinical signs scores (**D**) of each group are shown. The data are shown as means ± SD (error bars) (***, *p* < 0.001)

To examine the effect of miR-142t insertion into the PRRSV genome on viral replication and pathogenicity in vivo, we conducted an animal inoculation experiment using 4-week-old specific pathogens-free (SPF) pigs. We investigated the growth curve and pathogenicity of RvJX-miR-142t and its parental virus RvJXwn. The rectal temperature of pigs in the RvJXwn-infected group increased rapidly post-inoculation, and reached a peak (41.6 ℃) at 5 days post-inoculation (dpi) (Fig. 3A). The average rectal temperature of pigs in the RvJX-miR-142t infected group increased slowly, reaching its first peak with 40.5 ℃ at 11 dpi, then decreased, but two pigs had a fever again at 15 dpi. The average rectal temperature of pigs in the RvJX-miR-142t infected group was above 41.0 ℃ for three consecutive days from 16 to 18 dpi, and the highest temperature was 41.1 ℃. Compared with its parental virus, the temperature of pigs infected with RvJX-miR-142t had increased slower, and the body temperature response was reduced. Three pigs from each group were euthanized at 7 dpi, and all surviving pigs were euthanized and necropsied at 21 dpi. Six pigs in the RvJXwn infected group died during 5–13 dpi, and the mortality rate was 6/6. Two pigs in the RvJX-miR-142t infected group died on 19 dpi, and the mortality rate was 2/6 (Fig. 3B). Body weight of each pig from all groups was measured at 0 dpi, 7 dpi, 14 dpi, and 21 dpi, and the average daily weight gain (ADG) was calculated for 1–7 dpi, 8–14 dpi and 15–21 dpi, respectively (Fig. 3C). From 1–7 dpi, the ADG of pigs in the RvJXwn-infected group was –0.089 kg, while the average daily weight gain of pigs in RvJX-miR-142t infected group was 0.175 kg, significantly higher than that of the RvJXwn group (*p* < 0.001). Pigs infected with RvJXwn showed a rapid clinical onset with typical symptoms of HP-PRRS such as fever, anorexia, depression, dyspnea, tremors, paralysis, and eventually death. In contrast, all pigs in the RvJX-miR-142t infected group showed mild symptoms, such as rhinorrhea and sneezing before 15 dpi. Two pigs were depressed with severe clinical symptoms and died at 19 dpi. The clinical sign scores showed that clinical symptoms of pigs in the RvJX-miR-142t infected group were significantly lower than those in the parental virus RvJXwn infected group (Fig. 3D). The data indicated that RvJX-miR-142t was significantly attenuated in vivo, especially before 15dpi.

### The replication ability of RvJX-miR-142t was reduced significantly *in vivo*

**Fig. 4:**
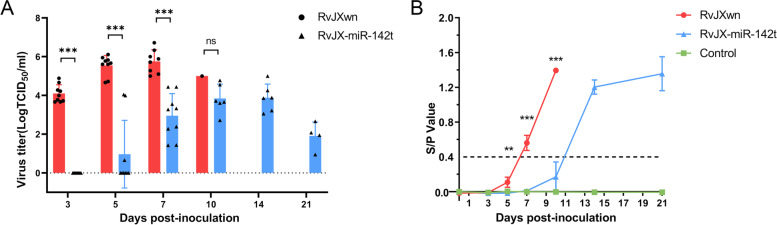
Viremia (**A**), and PRRSV-specific antibody kinetics (**B**) of pigs. The data are shown as means ± SD (error bars). Asterisks indicate a significant difference in the virus titers between RvJXwn and RvJX-miR-142t. (ns, *p* ≥ 0.05; **, *p* < 0.01; ***, *p* < 0.001)

The viremia of inoculated animals was examined using a microtitration infectivity assay (Fig. 4A). At 3 dpi, the average virus titer of the RvJXwn-infected group had reached 10^4^ TCID_50_/mL, whereas no PRRSV could be detected in the serum of any of the nine pigs in the RvJX-miR-142t-infected group. By 5 dpi, three pigs in the RvJX-miR-142t-infected group showed viremia, with two of them having a viral titer of around 10^4^ TCID_50_/mL. However the viral titer of the remaining pigs in this group was still undetectable, and the average titer was lower than that of the RvJXwn-infected group. At 7dpi, PRRSV could be detected in the serum of all pigs from the RvJX-miR-142t-infected group, and the virus titer among individuals within the group varied from 10^1.43^ TCID_50_/mL to 10^4.44^ TCID_50_/mL. This titer was significantly lower than that of the RvJXwn-infected group (*p* < 0.001). The average virus titer in the serum of all pigs in the RvJX-miR-142t infected group was about 10^4^ TCID_50_/mL at 10 dpi and 14 dpi. At 21 dpi, the virus titer in the serum of the RvJX-miR-142t group had decreased significantly. These results indicate that the proliferation ability of RvJX-miR-142t was significantly reduced in pigs, and virus replication was limited before 5 dpi but recovered in some pigs after 5 dpi. No virus was detected in the pigs from the negative control group.

Serum samples collected from the pigs were also tested for PRRSV-specific antibodies against its N protein (Fig. 4B). In the RvJXwn-infected group, one pig seroconverted at 5 dpi, six pigs seroconverted at 7 dpi, and the remaining pigs converted to positive at 10 dpi. In the RvJX-miR-142t infected group, only one pig was seroconverted at 10 dpi, and the seroconversion rate reached 100% at 14 dpi. The serology analysis showed that the RvJX-miR-142t infected group seroconverted later than the RvJXwn group, with a lower sample value/positive value ratio (S/P ratio) before 10 dpi. All pigs in the control group remained serologically PRRSV negative throughout the entire period. These results of PRRSV N protein antibody levels further indicated that RvJX-miR-142t had a lower proliferative capability in pigs, compared to its parental backbone virus RvJXwn.

### RvJX-miR-142t caused significantly less severe lung lesions compared with RvJXwn

**Fig. 5:**
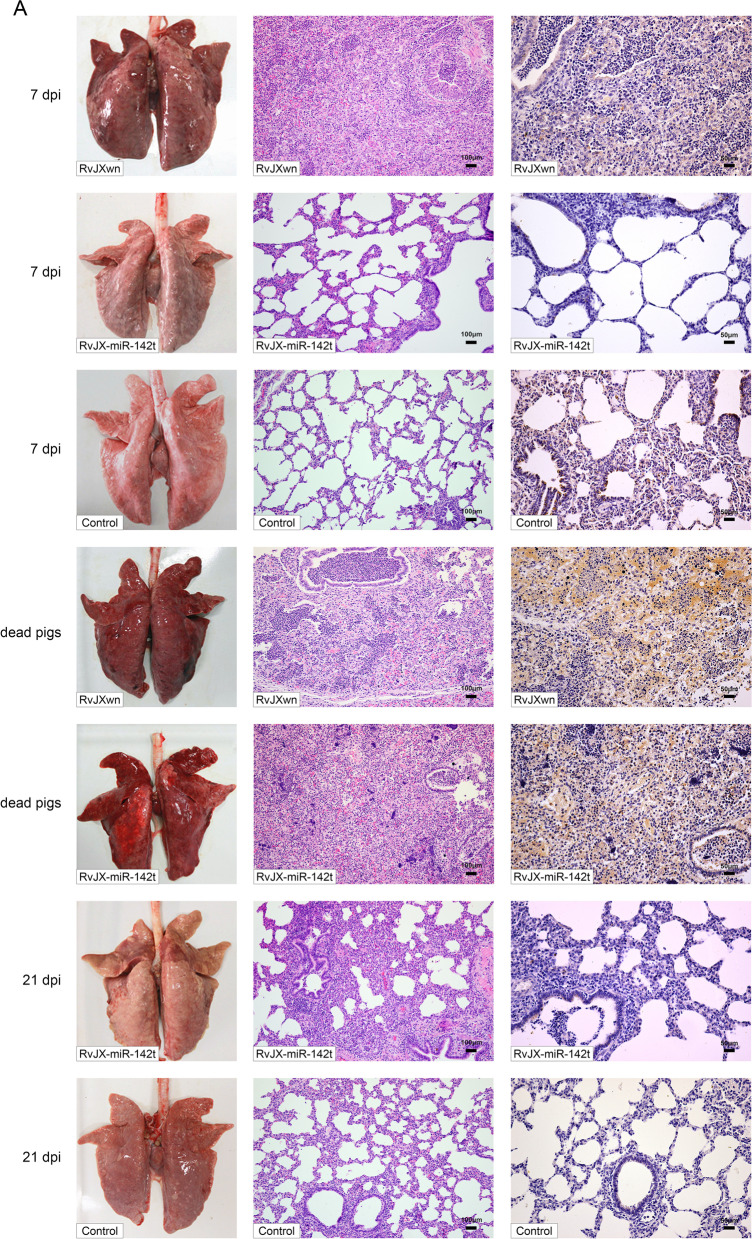

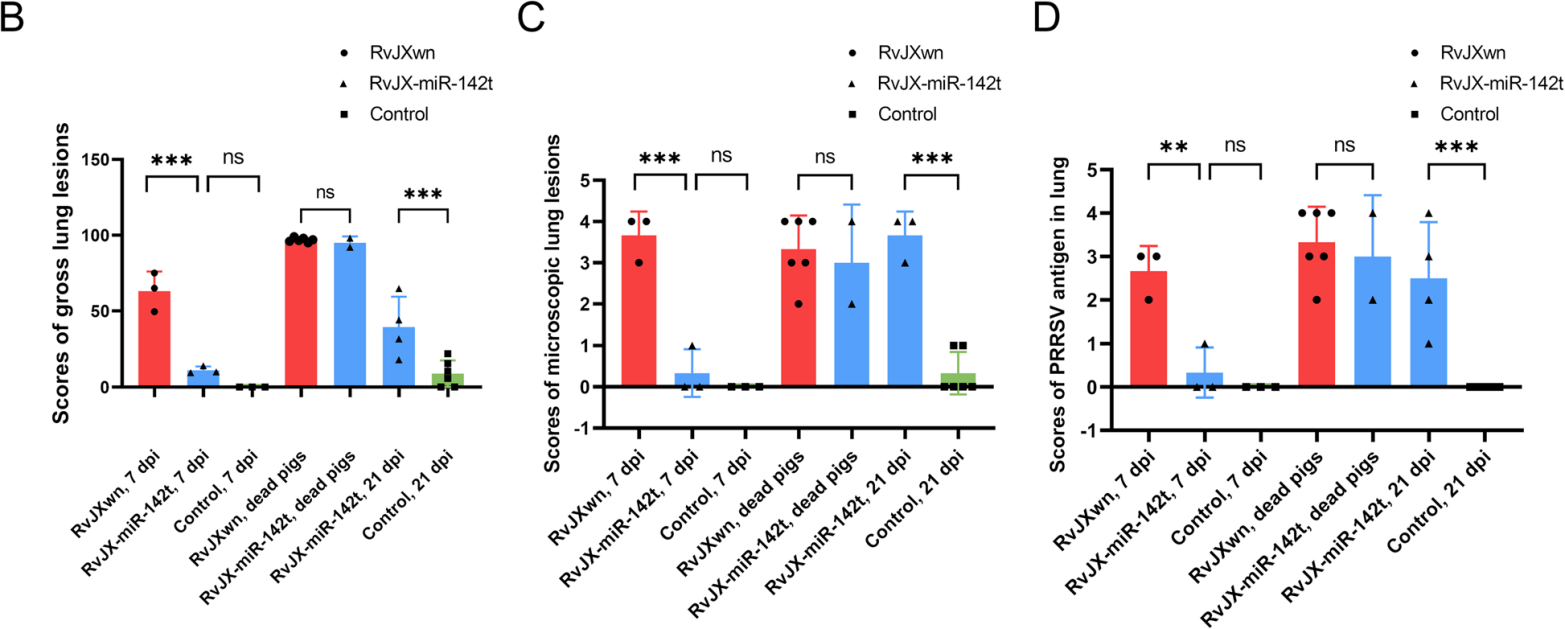
Lung lesions and immunohistochemistry examination. Representative pictures of gross and microscopic lung lesions and (**A**) are presented. And the scores of gross lung lesions (**B**), microscopic lung lesions (**C**), and PRRSV antigen in the lung (**D**) were shown as means ± SD (error bars), (ns, *p* ≥ 0.05; **, *p* < 0.01; ***, *p* < 0.001)

At 7 dpi, three pigs were randomly selected from each group and euthanized for necropsy. The lungs of pigs infected with RvJX-miR-142t showed scattered lesions, slight edema, and mild interstitial pneumonia. In contrast, the lungs from the RvJXwn-infected group showed typical interstitial pneumonia lesions as highly pathogenic strain infection. These lesions included severe congestion, edema, consolidation, and a mottled and tan appearing. The lungs of pigs in the control group appeared healthy (pink color) with no macroscopic lesions (Fig. 5A). Pigs died in the two inoculation groups were dissected, and the gross lung lesions were scored. There was no significant difference in the lung lesions between these two groups, and they showed typical interstitial pneumonia lesions such as congestion, edema, consolidation, and hemorrhagic lesions (Fig. 5B). At the end of the experiment (21 dpi), the remaining four pigs in the RvJX-miR-142t infected group, and all six pigs in the control group had survived. They were euthanized for necropsy and gross lung lesion scoring. The lungs of pigs in the RvJX-miR-142t infected group showed scattered lesions, slight edema, and mild interstitial pneumonia. These lesions were mainly concentrated in the apical lobe and heart lobe of the left and right lungs, and they were mild in the diaphragmatic lobe.

After hematoxylin and eosin (H&E) staining, ﻿the microscopic lung lesions were assessed, and the distribution of PRRSV antigen in the lungs was examined by immunohistochemistry (IHC) analysis with PRRSV N-specific monoclonal antibody (mAb) (Fig. 5A). All dead pigs in infected groups presented severe interstitial pneumonia with complete or extensive disappearance of lung structure, hemorrhage, thickening of the interlobular septa. Inflammatory cells and necrotic debris were infiltrate into both the alveolar spaces and bronchioles (Fig. 5A). The result of IHC showed that lungs of dead pigs were filled with PRRSV-positive signals generally located in the alveolar and septal macrophages around the bronchia, bronchioles, and alveolar septa (Fig. 5A). The mean histopathological and immunohistochemical scores were close to 4, with no significant difference between the RvJX-miR-142t and RvJXwn groups (Fig. 5C and D). For the surviving pigs at 7 dpi, lungs from RvJX-miR-142t infected pigs had scores similar to those from the control group, and lungs from the RvJXwn group presented moderate microscopic lesions with partial disappearance of lung structure, minor hemorrhage, and inflammatory cells and necrotic debris within both the alveolar spaces and bronchioles. The lesion score of the RvJX-miR-142t infected group was significantly higher than that of the control group at 21 dpi. These results suggested that RvJX-miR-142t had delayed and less severe lung lesions in pigs.

### The deletion of the miR142 target sequence resulted in the reversion of virus proliferation

**Fig. 6:**
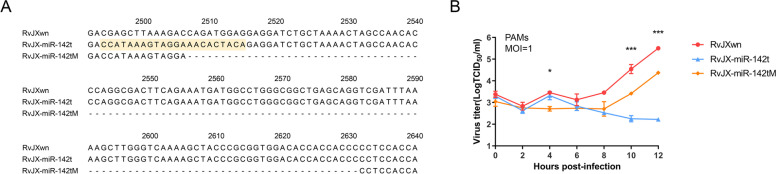
Characteristics of the RvJX-miR-142tM. (**A**) Sequences alignment of the miR-142 target sequence inserted region in PRRSV nsp2. The miR-142 target sequence is in the yellow box. Dashes indicate deleted amino acids compared to RvJX-miR-142t. (**B**) The growth kinetics of RvJX-miR-142tM. The data are shown as means ± SD (error bars). Asterisks indicate a significant difference in the virus titers between RvJX-miR-142t and RvJX-miR-142tM. (*, *p* < 0.05; ***, *p* < 0.001)

To find out why RvJX-miR-142t can still proliferate after 5 dpi and have a late onset of clinical symptoms, serum samples collected from six pigs in the RvJX-miR-142t infected group at 14 dpi were submitted for PRRSV isolation and sequencing. The PRRSV genome was directly extracted from sera samples and sequenced, followed by alignment analysis. The dominant PRRSV strains in the serum of the six pigs named RvJX-miR-142tM all had the same 126 bases deletion (located in 2506–2631 nt of the full-length sequence of RvJX-miR-142t), and the deletion site started from the last 9 bases of miR-142t (Fig. 6A). To investigate if the deletion caused the escape from inhibition of miR142, the isolated strain RvJX-miR-142tM was inoculated on PAMs. Its growth kinetics were then compared with RvJX-miR-142t and RvJXwn. It was observed that the virus titer of RvJX-miR-142t continually reduced after 4 hpi; however, the virus titer of RvJX-miR-142tM started to increase after 8 hpi. Even though it showed a lower virus titer than that of RvJXwn, they already presented a similar trend (Fig. 6B). The results indicated that the RvJX-miR-142tM can escape from the inhibitory effect of miR-142 in PAMs, leading to reversion of proliferation *in vitro*. We highly suspected that this was the main reason for virulence reversion observed *in vivo*.

## Discussion

PRRSV is an pathogen of economic importance that causes many challenges for pigs. To date, there is no fully satisfactory vaccine available on the market that balances safety and efficacy. The killed PRRSV vaccine is safe, but it can’t provide effective protection by only inducing humoral immunity. Meanwhile, PRRSV MLV excels at providing great homologous protection, with drawbacks such as recombination with field strains and reversion to virulence, as well as partially inhibiting the host immune response. The ideal vaccine strain should be able to “slightly” infect the host cells and induce a cellular immune response, but its replication in host cells could be suppressed to reduce its virulence. Compared with killed vaccine, this strategy might induce a cell-mediated immune response to provide the critical factor for protection. At the same time, its proliferation in the cell-culture system is unlimited to reduce the cost of production. Therefore, exclusively inhibiting PRRSV replication in its target cells, such as PAMs, is a valuable strategy for viral attenuation.

It has been reported that tissue-specific miRNAs can control viral tropism by targeting specific regions of the virus genome to restrict viral replication exclusively in this cell population. For example, the hematopoietic-cell-specific miRNA miR-142-3p has been found to restrict the replication of the mosquito-borne North American eastern equine encephalitis virus (EEEV) in myeloid-lineage cells by binding to sites in the 3’ non-translated region of its RNA genome [27]. Furthermore, the target sequences can also be inserted into the viral genome by using reverse genetic operation. To study whether the replication in antigen-presenting cells (APCs) is required to induce the antiviral immune response of the influenza virus (IAV), the target sequence of miR-142 was inserted into its nucleoprotein to effectively silence virus transcription in APCs [28]. A similar strategy has also been used to restrict the tropism of dengue virus (DENV) to identify whether non-hematopoietic cells can serve as virus reservoirs for DENV [23]. Based on our unpublished data, the expression level of miR-142 in PAMs is stable and not regulated by PRRSV infection. Therefore, the target sequence of miR-142 was used in this study to be inserted into the PRRSV genome to specifically inhibit PRRSV replication in PAMs.

As a highly diverse and variable virus, it's possible to engineer the PRRSV genome with point mutations, insertions, deletions, replacements, or recombinations [29]. The nsp2 coding region is regarded as the most hypervariable region of the PRRSV genome, and many viruses with modifications in this region have been successfully rescued. To increase the possibility of rescuing a viable recombinant virus, the target sequence of miR-142 was inserted here. According to previous studies, the direct insertion of exogenous gene fragments into the nsp2 coding region may cause gene mutations or deletions during passage in cells, while insertion of exogenous gene fragments by replacement can achieve stable expression [30, 31]. Therefore, in this study, the isometric gene fragment in the hypervariable region of PRRSV nsp2 was replaced by the target sequence of miR-142 to construct the chimeric virus. As we expected, the rescued virus RvJX-miR-142t remained its normal proliferation in MARC-145 cells, but its replication was inhibited in MARC-145 with overexpressed miR-142 mimic and significantly decreased in PAMs. Further animal inoculation experiments showed that RvJX-miR-142t infected pigs had weakened clinical symptoms, lower viremia titers, lighter lung lesions, and significantly reduced mortality, compared with the pigs in RvJXwn group. Especially during the early 7 days post-inoculation, there is no significant difference between the RvJX-miR-142t infected group and the negative control group, in terms of ADG, scores of clinical signs, and scores of gross or microscopic lung lesions. These data indicated this strategy worked for PRRSV.

However, multiple indications of virulence reversion were observed in RvJX-miR-142t infected group after 7 dpi. The viral titers of RvJX-miR-142t were first identified in three pigs at 5 dpi, and all pigs in this group were detected as PRRSV positive at 7 dpi. In addition, we also observed reduced ADG, increased scores of clinical signs, increased gross or microscopic lung lesion scores, and increased mortality in the RvJX-miR-142t-infected group, indicating that the modified virus has recovered its proliferation capability in pigs in the later term. Following sequencing results confirmed the conjecture that the RvJX-miR-142t partially lost the target sequence of miR-142, and the isolated virus (named RvJX-miR-142tM) recovered its replication ability in PAMs again. PRRSV is characterized by its high frequency of mutation, among known RNA viruses. Previous research has shown that selective mutations from the vaccine virus genome to virulent strain occurred during the proliferation in pigs [32, 33]. It is not surprising that RvJX-miR-142t lost 126 bases in pigs and became virulent again in our study. Although the pathogenicity of RvJX-miR-142t in pigs has significantly decreased, the reversion of virulence impedes its potential to be developed as a vaccine. To obtain a high hereditary stability chimeric virus, multiple copies of the miR-142 target sequence were inserted into the PRRSV genome between ORF1b and ORF2a in our later study (unpublished data). After purification by plaque assay, we obtained a chimeric virus containing 3 copies of miR-142t between ORF1b and ORF2a, named RvJX-miR-142-3t. The proliferation ability of the chimeric virus was significantly lower than that of the RvJXwn, and it cannot proliferate in PAMs. Animal experiments were carried out similarly to this study. Results showed that RvJX-miR-142-3t can lead to less severe and delayed clinical signs. RT-PCR and sequence analysis showed that the inserted miR-142-3t in the chimeric virus had varying degrees of base deletion since 3 dpi. Considering that foreign fragments inserted into the area do not interrupt viral viability, it is easy to lose them under selective pressure from the host. Therefore, finding a new RdRP with increased fidelity might provide some possibility to solve this problem.

Taken together, the strategy of inserting a target sequence of hematopoietic-specific miR-142 into the genome of PRRSV can limit viral proliferation in its target cells PAMs and reduce pathogenesis in pigs. Our study has provided a new strategy for PRRSV vaccine development; however, risks of virulence reversion remained. Further studies are needed to increase the stability of the modified viruses before they can be used as vaccines clinically.

## Materials and Methods

### Cells, viruses, and plasmid

Primary PAMs were prepared from 4-week-old specific-pathogen-free (SPF) landrace pigs and cultured in RPMI 1640 medium (Thermo Fisher Scientific) with 10% FBS, as previously described [34, 35]. For PRRSV proliferation and titration, MARC-145, an MA104-derived monkey kidney cell line, was cultured in Dulbecco’s modified Eagle’s medium (DMEM) (Thermo Fisher Scientific) with 10% fetal bovine serum (FBS) (Gibco) and penicillin (50 U/mL)-streptomycin (50 μg/mL). BHK-21 cells used for transfection of full-length cDNA were cultured by the same method as MARC-145 cells. All cells were cultured at 37 °C in a humid 5% CO_2_ incubator. The full-length infectious cDNA clone plasmids (pWSK-JXwn) of HP-PRRSV JXwn06 (GenBank accession number EF641008), the pJET-Blunt vector, and the rescued viruses (RvJXwn) were stored in our laboratory [3].

### Construction of full-length cDNA clones with miR-142 target sequence insertion

The strategy for inserting the miR-142 target sequence into the full-length cDNA clones of PRRSV is illustrated in Fig. 1. The 22nt miRNA-142 target sequence (CCATAAAGTAGGAAACACTACA) was used to replace a part of nsp2 hypervariable region with the same length (2493 nt to 2514 nt). To construct this chimeric PRRSV full-length infectious clone, the genome of HP-PRRSV JXwn06 was initially divided into two fragments. The enzyme cleavage site Not I, SP6 polymerase promoter, and one non-templated G residue were added to the forward primer for fragment A. And fragment A was amplified by integrating two overlapping 5’-Not I and 3’-BstB I PCR segments using fusion PCR. The PCR product was gel purified and subcloned into a pJET-Blunt vector. The constructed plasmid pJET-JA-miR-142t was sequenced to confirm the correct insertion of the target sequence and no additional mutation was introduced during PCR. Then the fragment was excised by using restriction enzymes Not I and BstB I, and ligated into excised plasmid pWSK-JXwn by T4 ligase (Promega). After that, the product was transformed into Escherichia coli DH5α cells and grown overnight at 37 °C in the presence of ampicillin. The full-length cDNA clone, plasmid pWSK-JX- miR-142t, was extracted and sequenced. The sequences of the primers used for construction are listed in Table 1.

**Table 1:** Primers used for construction and identification of pWSK-JX-miR-142t

Primer^a^	Sequence (5’-3’)^b^	Position^c^	Purpose
miR-142t-F	CCATAAAGTAGGAAACACTACAGAGGATCTGCTAAAACTAGCC	2493–2535	Amplification of miR142t
miR-142t-R	TGTAGTGTTTCCTACTTTATGGTCGAGCCCGCTCGGCAGCACG	2472–2514	Amplification of miR142t
JAF (SP6)	**GCGGCCGC**GCGATTTAGGTGACACTATA*G*ATGACGTATAGGTGTTGGCTCTATG **(NotI)**	1–25	Fragment clone
JAR	GGC**TTCGAA**ATTTGCCTGATCTT **(BstBI)**	4804–4826	Fragment clone
miR-142t	CCATAAAGTAGGAAACACTACA	2493–2514	Sequencing
W1F	ATGACGTATAGGTGTTGGCTCT	1–22	Sequencing
W1R	TACTCTTTCAGGAAGGGTGG	1556–1575	Sequencing
W2F	ACGCTCTGGTGCGACTACTA	1362–1381	Sequencing
W2R	AGGTTGTTCGGTTGTCTGATT	2233–2253	Sequencing
W3F	CCTCCGTGGCGCAACAAGTCTTG	2115–2137	Sequencing
W3R	CGATGATGGCTTGAGCTGAGTAT	3156–3178	Sequencing
W4F	TGAGCCTCTGGATTTGTCTGC	2952–2972	Sequencing
W4R	GGCGATCTCATTAGGAGCAGTT	4308–4329	Sequencing
W5F	TGCTTAGGCTTGGCATTGTTG	4214–4234	Sequencing
W5R	ACGGTGTTCAGTGAGGGCTTT	5544–5564	Sequencing

^a^F denotes forward PCR primer; R denotes reverse transcription or reverses PCR primer

^b^Restriction sites introduced by PCR are shown in boldface and specified in parentheses at the end of the sequence. And a viable chimeric virus designed as RvJX-miR-142t was successfully rescued

^c^Numbers refer to nucleotide positions within the genome of JXwn06 (GenBank accession no: EF641008) as indicated. The underline denotes the miR142 target sequence (miR142t) at the first two lines and denotes the SP6 promoter followed by a nongenomic G (italicized) at the third line

### Recovery of chimeric virus

The chimeric full-length cDNA clone of RvJX-miR-142t was linearized by cleavage with restriction enzyme PacI at the 3’end of the ploy-A tail. The capped RNAs were transcribed with SP6 RNA polymerase by using a mMessage high-yield capped RNA transcription kit (Ambion, Austin, TX) according to the manufacturer's instructions. The RNAs were quantified by spectrophotometry and then transfected into BHK-21 cells by using Invitrogen DMRIE-C reagent(Invitrogen life technologies) according to the manufacturer's instructions [3]. The supernatant of cell culture was obtained at 24 h post-transfection and then serially passaged on MARC-145 cells. The cytopathic effect (CPE) was observed daily. The rescued viruses were confirmed by an immunofluorescence assay (IFA) using PRRSV N protein-specific monoclonal antibodies (prepared and preserved by our laboratory).

### In vitro stability and growth kinetics of the rescued viruses

To assess the replication stability of the rescued viruses, the viruses were serially passaged (P1 to P10) in MARC-145 cells, and the PRRSV genome with miR142-t in the viruses was sequenced at P5 and P10. To analyze the in vitro growth characteristics, the monolayers MARC-145 or primary PAMs were individually infected with rescued RvJX-miR-142t virus and RvJXwn at the same multiplicity of infection (MOI) of 0.01 or 1. To investigate whether the miRNA target of RvJX-miR-142t was effective, the miR142 mimic was transfected into MARC-145 cells by using Lipofectamine 2000 reagent (Invitrogen), before being inoculated with RvJX-miR-142t or RvJXwn (MOI = 0.01), and the NC were also set as controls. Virus titers in cell cultures were determined by a microtitration infectivity assay and recorded as TCID_50_ per milliliter by using the Reed-Muench method. Briefly, cells were prepared in 96-well plates and inoculated with virus suspensions (100 μl/well), which were prepared by serial tenfold dilution with DMEM with 5% FBS. Plates were incubated for 48 h, and the virus was determined by IFA. All tests were independently repeated three times.

### Animal trials for pathogenicity analyses of the rescued chimeric viruses

To evaluate the pathogenicity of the chimeric virus, twenty-seven 6-week-old SPF Landrace pigs were obtained from the Beijing Center for SPF Swine Breeding and Management. All pigs were confirmed to be negative for PRRSV nucleic acid and antibody. The animals were randomly divided into three groups (nine pigs per group), and each group was separately raised in isolated rooms in the animal facility of China Agricultural University (CAU). The pig in each group was intranasally inoculated with 2 mL of virus deluded to 10^5^ TCID_50_ (RvJX-miR-142t or RvJXwn) or 2 mL supernatant of MARC-145 cells as the negative control, respectively. The clinical symptoms, including rectal temperature, respiratory disease scores and were recorded daily as previously described, and the average daily weight gain (ADG) was calculated by weighting the pigs weekly as previously described [36]. Serum samples were also collected on 3, 5, 7, 10, 14, and 21 dpi for the titration of viral loads by a microtitration infectivity assay and detecting antibodies specific for PRRSV N protein by using the IDEXX Herdchek PRRS 2XR ELISA kit (IDEXX). Three pigs for each group were randomly selected, euthanized, and necropsied at 7 dpi and all the survived pigs were euthanized and necropsied at 21 dpi. Necropsy, gross and microscopic pathological evaluation, as well as immunohistochemistry (IHC) examinations, were immediately performed once the inoculated pigs died during the experiment, and after the euthanize at 7 and 21 dpi as previously described [36, 37].

### Characteristics of genomic sequence and proliferation of chimeric PRRSV after inoculation

To identify whether the genomic sequence of RvJX-miR-142t has changed during infecting pigs, the genome of PRRSV was extracted and reverse transcription from 6 RvJX-miR-142t infected pigs’ serum at 14 dpi by using QIAamp Viral RNA Mini Kit (Qiagen) and M-MLV reverse transcription enzyme (Promega Corporation), followed by PCR and sequencing. The sequences were analyzed by Lasergene and Geneious Prime in this study. On the other hand, the RvJX-miR-142t infected pigs’ serum at 14 dpi was submitted for virus isolation. Three days later, the supernatants were harvested and stored at –80℃, and the isolated virus was named RvJX-miR-142tM. The growth kinetics of RvJX-miR-142t, RvJX-miR-142tM, and RvJXwn were examined as described above.

### Statistical analysis

Data were expressed as means ± standard deviations. The significance of the variability among the animal trials was determined by a two-way analysis of variance or two-tailed unpaired t-test using GraphPad Prism (version 5.0) software. Differences were considered statistically significant at a value of *p* < 0.05 and extremely significant at a value of *p* < 0.01 or *p* < 0.001.

## Data Availability

Data will be shared upon request by the readers.
